# Cellular export of sugars and amino acids: role in feeding other cells and organisms

**DOI:** 10.1093/plphys/kiab228

**Published:** 2021-05-20

**Authors:** Ji-Yun Kim, Eliza P -I Loo, Tin Yau Pang, Martin Lercher, Wolf B Frommer, Michael M Wudick

**Affiliations:** 1 Institute for Molecular Physiology and Cluster of Excellence on Plant Sciences (CEPLAS), Heinrich-Heine-University Düsseldorf, Düsseldorf 40225, Germany; 2 Institute for Computer Science and Department of Biology, Heinrich-Heine-University Düsseldorf, Düsseldorf 40225, Germany; 3 Institute of Transformative Bio-Molecules (WPI-ITbM), Nagoya University, Chikusa, Nagoya 464-8601, Japan

## Abstract

Sucrose, hexoses, and raffinose play key roles in the plant metabolism. Sucrose and raffinose, produced by photosynthesis, are translocated from leaves to flowers, developing seeds and roots. Translocation occurs in the sieve elements or sieve tubes of angiosperms. But how is sucrose loaded into and unloaded from the sieve elements? There seem to be two principal routes: one through plasmodesmata and one via the apoplasm. The best-studied transporters are the H^+^/SUCROSE TRANSPORTERs (SUTs) in the sieve element-companion cell complex. Sucrose is delivered to SUTs by SWEET sugar uniporters that release these key metabolites into the apoplasmic space. The H^+^/amino acid permeases and the UmamiT amino acid transporters are hypothesized to play analogous roles as the SUT-SWEET pair to transport amino acids. SWEETs and UmamiTs also act in many other important processes—for example, seed filling, nectar secretion, and pollen nutrition. We present information on cell type-specific enrichment of SWEET and UmamiT family members and propose several members to play redundant roles in the efflux of sucrose and amino acids across different cell types in the leaf. Pathogens hijack SWEETs and thus represent a major susceptibility of the plant. Here, we provide an update on the status of research on intercellular and long-distance translocation of key metabolites such as sucrose and amino acids, communication of the plants with the root microbiota via root exudates, discuss the existence of transporters for other important metabolites and provide potential perspectives that may direct future research activities.

## Teaser

An update on intercellular and long-distance translocation of sugars and amino acids, including plant-root microbiota communication, other metabolite transporters is provided, and perspectives are discussed.

## Introduction

Cells can secrete specific compounds for various functions, for example, disposal, protection from osmotic damage, feeding of other cells—either a neighboring cell in the same organism or cells from other organisms—or solute distribution in multicellular organisms, and defense. A well-studied example is *Corynebacterium glutamicum*, which effectively secretes glutamate and is therefore used for the industrial production of glutamate (Nakayama et al., 2018). *Corynebacterium glutamicum* secretes glutamate via a mechanosensitive efflux transporter. Many bacteria secrete valine into their biofilms where it serves as an antibiotic (Valle et al., 2008). This review focuses on processes in which major metabolites, in particular sugars and amino acids, are secreted from plant cells. Key physiological aspects discussed here relate to the distribution of assimilates in plants, as well as to the exchange of metabolites with other organisms, in particular nectar secretion and feeding of beneficial and pathogenic microbes. This review highlights families of transporters for metabolites—sugars and amino acids—and their role in carbon and nitrogen allocation: SWEETs and UmamiTs, as well as additional transporters for other metabolites and their roles in physiology, pathogenesis, and symbiosis.

**Advances**

- SWEET sugar transporters and UmamiT amino acid transporters are expressed in specific cell types that play roles in secretory functions.
- Amino acid metabolism in two phloem cell types, phloem parenchyma and CCs, are distinct, indicating the metabolism in the two phloem cell types may shape the relative amino acid composition of the phloem sap.
- Numerous SWEETs across different plant families are induced during arbuscular mycorrhizal fungus and rhizobial symbiosis, implicating SWEETs in symbiotic nutrition.
- Metabolomic studies reveal region dependent root exudation under various growth conditions.

## SWEET and UmamiT transporters: evolution and structure

Members of the SemiSWEET-SWEET sugar transporter superfamily had originally been described as homologs of *Medicago truncatula NODULIN 3* (*Mt*N3), based on the observation that its transcript levels increased during nodulation (Gamas et al., 1996). SWEETs belong to an ancient family with members present already in Archaea. Plant genomes typically contain approximately 20 *SWEET*s with two conserved PQ-loop repeats (Table 1; Supplemental Figure S1; Supplemental Table S1).

**Table 1:** Transporters for metabolites with potential roles in cellular efflux discussed in this study

Transporter	Family	Super Family	Conserved Domain(s)	PFAM	Interpro	TCDB	PDB
SWEET (*Mt*N3-like)	SWEET/Semi SWEET	SWEET	Sugar efflux transporter, PQ-loop repeat	PF04193–PF03083	IPR004316	2.A.123	5CTH, 5XPD, 5CTG
UmamiT (*Mt*N21-like)	P-DME	DMT	EamA-like repeat	PF00892	IPR000620	2.A.7.4	5I20^a^
MATE	MATE	MviN	MatE	PF01554	IPR002528	2.A.66	5Y50
ALMT	ArAE	UspB	–	PF11744	IPR020966	9.A.85	N.D.

Abbreviations: ABC, ATP-binding cassette; ArAE, aromatic acid exporter; *Mt*N3 or *Mt*N21-like, *Medicago truncatula* nodulin 3 or 21- like; MviN, mouse virulence N; N.D., not determined; PDB, protein database; P-DME, plant drug/metabolite exporter; PFAM, protein family; TCDB, transporter classification database; UspB, universal stress protein-B.

^a^ Bacterial homolog.

UmamiT amino acid transporters had originally been described as *Mt*N21-like nodulins (Dinkeloo et al., 2018). They belong to the plant-specific branch of the Drug/Metabolite Exporter (P-DME) family, which is part of the larger family of drug/metabolite transporters (DMT), and which shares sequence homology with members of the five transmembrane-domain Bacterial/Archaeal transporter (BAT) family. Prokaryotic DMT paralogs function as amino acid efflux transporters, for example, the *Escherichia coli* O-acetyl-serine/cysteine exporter *EamA* (Franke et al., 2003). UmamiTs contain two EamA-like domains (Table 1). Plants typically have approximately 50 UmamiT paralogs per haploid genome (Table 1; Supplemental Figure S2; Supplemental Table S2).

**Figure 1:**
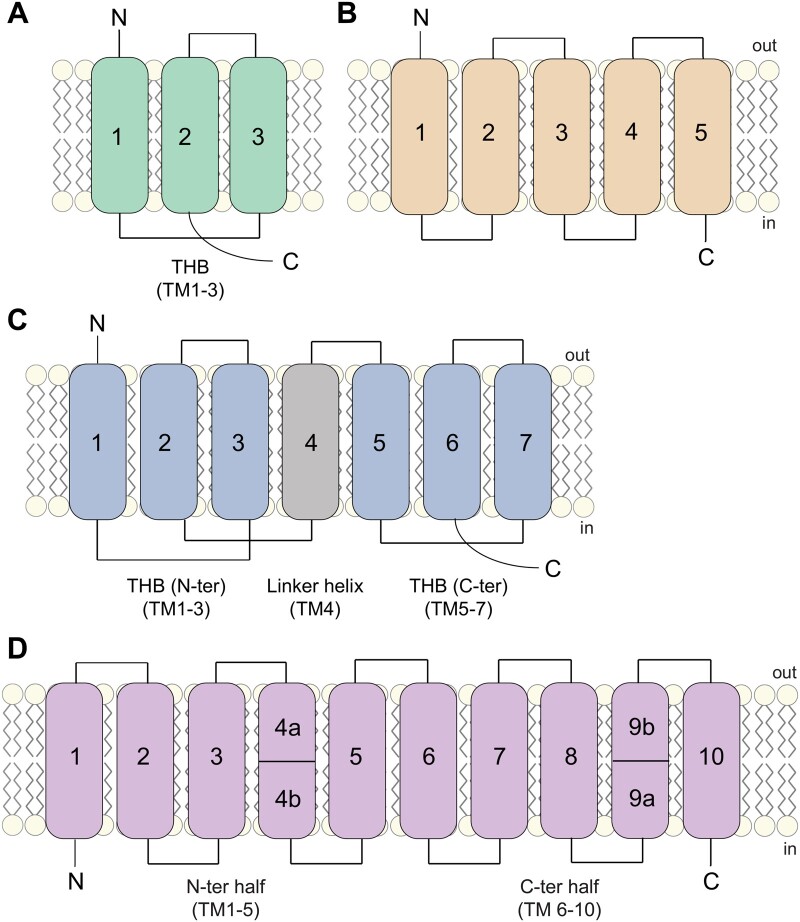
Topology of SemiSWEET, SWEET, SemiUmamiT and UmamiT. A, Bacterial SemiSWEET unit comprised of a triple helix bundle (green). B, Bacterial SemiUmamiT topology based on bioinformatic analyses (Aramemnon) and the structure of BAT1 (Jack et al., 2001). C, Topology of eukaryotic SWEETs comprised of two triple helix bundles (light blue) fused via an additional linker helix (gray). D, Predicted topology for UmamiT based on bioinformatic analyses (Aramemnon) and the structure of the amino acid exporter YddG (Tsuchiya et al., 2016). TM, transmembrane domain; THB, triple helix bundle. Numbers indicate transmembrane helices (represented as boxes).

Structures of SWEETs, SemiSWEETs, their ancestral prokaryotic homolog, and a distant UmamiT homolog have been resolved by X-ray crystallography (Xu et al., 2014; Tao et al., 2015; Tsuchiya et al., 2016; Han et al., 2017; Latorraca et al., 2017; Figure 1, A–D). While eukaryotic SWEETs are composed of seven transmembrane helices, with an apparent parallel symmetry axis (3 + 1 + 3), prokaryotic SemiSWEETs are amongst the smallest known transporters with only three helices (Figure 1A). SemiSWEET pores are formed by parallel oriented dimers, while eukaryotic SWEETs contain a central fourth transmembrane helix that orients the second repeat in the same orientation as in dimeric SemiSWEETs (Xu et al., 2014; Tao et al., 2015; Han et al., 2017; Figure 1, A and C). SemiSWEETs have two gates, and the transport cycle alternates between outside open, occluded and open inside conformations (Latorraca et al., 2017).

**Figure 2:**
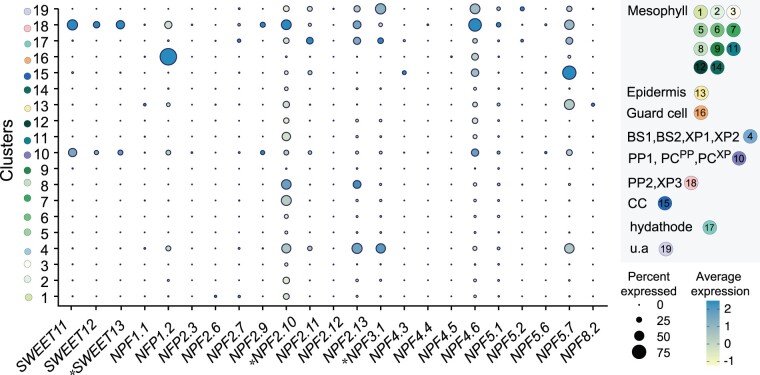
Leaf cell type specificity of GA transporters identified in heterologous system (Xenopus oocytes and yeast) and in planta (*). Dot plot showing transcript enrichment of GA transporters across 19 clusters of the leaf scRNA-seq data (Kim et al., 2021). The diameter of the dot indicates the percentage of cells in the cluster in which transcripts for that gene were detected, while the color of each dot represents the average log-scaled expression of each gene across all cells within a given cluster (see legend at lower right side). Cell types assigned to each cluster are indicated in the upper right panel. BS1, bundle sheath; BS2, bundle sheath cells enriched with photosynthetic processes, XP1 and XP2, xylem cells related with the bundle sheath; XP3, xylem cells enriched with vascular parenchyma markers; PC^XP^, procambium cells related to XP1; PC^PP^, procambium cells related to PP cells with transfer cell identity (PP1); u.a., unassigned. Note that NPF proteins transport additional substrates as reviewed in Corratgé-Faillie and Lacombe, 2017. The mRNA counts of *SWEET9*, *SWEET10*, *SWEET14*, *NPF5.3*, *NPF4.2*, *NPF4.1*, *NPF2.5*, *NPF2.4*, *NPF2.1* were not detected in the dataset (Kim et al., 2021). For detailed information about the dataset and description of the clusters/subclusters, refer to Kim et al. (2021).

UmamiTs are predicted to contain ten transmembrane helices, similar to their distant prokaryotic homolog YddG, which functions as an amino acid exporter (Tsuchiya et al., 2016; Figure 1D). Most DMTs consist of inverted structural repeats, which are basket-shaped and related by a two-fold pseudosymmetry, yielding a substrate binding cavity at the center (Tsuchiya et al., 2016). Similar to SemiSWEETs, the prokaryotic BATs may form “half” transporters with five transmembrane helixes (Figure 1B). UmamiTs may have arisen by intragenic duplication from such ancestral transporter domains (Jack et al., 2001). Interestingly, the genome of Arabidopsis (*Arabidopsis thaliana*) also seems to code for “half”- or SemiUmamiTs. For instance, UmamiT43 is predicted with five transmembrane segments (http://aramemnon.uni‐koeln.de), but has not yet been functionally characterized.

## SWEET and UmamiT substrates

The activity of SWEETs was identified through a screen of polytopic membrane proteins with unknown functions coexpressed with genetically encoded glucose or sucrose sensors in human embryonic kidney (HEK293T) cells (Chen et al., 2010, 2012). It was hypothesized that human cells, which are cultured in media with a neutral pH, and which lack plasma membrane H^+^-ATPases, may provide favorable conditions for identifying sucrose efflux transporters that might function as uniporters or sucrose/H^+^ antiporters (Fieuw and Patrick, 1993). SWEETs, similar as SemiSWEETs, can transport hexoses and/or the disaccharide sucrose (Table 2, Supplemental Figure S1; Chen et al., 2010, 2012; Xu et al., 2014; Tao et al., 2015). Phylogenetically, Arabidopsis SWEET members fall into four clades in which clade 3 members preferably mediate sucrose transport (Supplemental Figure S1). In addition to sugar transport, several SWEETs are capable of transporting gibberellic acid (GA), which at first sight, neither resembles glucose nor sucrose (Kanno et al., 2016; Morii et al., 2020; Table 2). Notably, GA biosynthesis pathway genes are enriched in phloem cell types where *AtSWEET11-13* and several other GA transporters, such as *AtNPF4.6*, are enriched (Figure 2; Kim et al., 2021). Although all characterized clade III SWEETs are plasma membrane-localized, other members (i.e. clades II, IV) were also detected in vacuolar and ER membranes (Table 2, Supplemental Figure S1).

**Table 2:** SWEETs in Arabidopsis

Gene Name (Alternative Name)	Substrate(s)	Locali-zation	Physiological Role	Reference
*SWEET1*	Glucose	PM	N.D.	Chen et al., 2010
*SWEET2*	2-DOG	TP	Resistance to *Pythium spp*	Chen et al., 2015a; Veillet et al., 2017; Sellami et al., 2019; Desrut et al., 2020
*SWEET3*	2-DOG	N.D.	N.D.	Chen et al., 2015b; Desrut et al., 2020; Liao et al., 2020
*SWEET4*	Glucose	PM	Sugar supply to the axial tissues, freezing and drought tolerance and nonhost resistance	Chen et al., 2010; Liu et al., 2016; Desrut et al., 2020; Liao et al., 2020
*SWEET5* (*VEX1*)	Glucose	ND	Possibly transport of sugars in vegetative cell of pollen grains	Engel et al., 2005; Borges et al., 2008; Chen et al., 2010; Borges et al., 2012; Liao et al., 2020
*SWEET6*	2-DOG	ER	N.D.	Chen et al., 2010; Lee et al., 2011; Chen et al., 2015b
*SWEET7*	Glucose	N.D.	N.D.	Chen et al., 2010; Liao et al., 2020
*SWEET8* (*RPG1*)	Glucose	PM	Microspore development, pollen mitosis, primexine deposition, tapetum efflux	Drakakaki et al., 2006; Chen et al., 2010; Sun et al., 2013; Veillet et al., 2017; Liao et al., 2020
*SWEET9*	Sucrose, weak glucose, GA	PM, TGN	Nectar secretion	Lin et al., 2014; Kanno et al., 2016; Durand et al., 2018
*SWEET10*	Sucrose, GA	N.D.	Floral transition	Chen et al., 2012, 2015a; Kanno et al., 2016; Durand et al., 2018; Andrés et al., 2020; Desrut et al., 2020)
*SWEET11*	Sucrose, glucose, fructose, GA	PM	Efflux of sucrose from PP for phloem loading, embryo nutrition, vascular development, freezing tolerance, salicylic acid-mediated defense response	Chen et al., 2012, 2015c; Eom et al., 2015; Le Hir et al., 2015; Durand et al., 2016, 2018; Kanno et al., 2016; Gebauer et al., 2017; dos Anjos et al., 2018; Walerowski et al., 2018; Dinant et al., 2019; Sellami et al., 2019; Desrut et al., 2020; Huang et al., 2020; Wei et al., 2020; Zhao et al., 2020; Fichtner et al., 2021
*SWEET12*	Sucrose, glucose, fructose, GA	PM	Efflux of sucrose from PP for phloem loading, embryo nutrition, vascular development, freezing tolerance, salicylic acid-mediated defense response	Chen et al., 2012, 2015c; Duan et al., 2014; Eom et al., 2015; Le Hir et al., 2015; Durand et al., 2016, 2018; Kanno et al., 2016; Gebauer et al., 2017; dos Anjos et al., 2018; Walerowski et al., 2018; Dinant et al., 2019; Sellami et al., 2019; Desrut et al., 2020; Huang et al., 2020; Wei et al., 2020; Zhao et al., 2020; Fichtner et al., 2021
*SWEET13* (*RPG2*)	Sucrose, GA	PM	Anther dehiscence, germination, seed development, vegetative growth, microspore development, pollen mitosis, primexine deposition, tapetum efflux	Durand et al., 2016, 2018; Kanno et al., 2016; Han et al., 2017; Sellami et al., 2019; Andrés et al., 2020; Zhao et al., 2020; Fichtner et al., 2021
*SWEET14*	Sucrose, GA	PM	Anther dehiscence, germination, seed development, vegetative growth	Durand et al., 2016, 2018; Kanno et al., 2016; Sellami et al., 2019; Andrés et al., 2020
*SWEET15* (*SAG29*)	Sucrose	PM	Embryo nutrition, accelerated senescence in overexpression lines	Chen et al., 2010, 2015c; Seo et al., 2011; Matallana-Ramirez et al., 2013; Qi et al., 2015; Durand et al., 2016, 2018; Gao et al., 2016; Rasheed et al., 2016; Zhao et al., 2016; Gebauer et al., 2017; Kihira et al., 2017; Sellami et al., 2019; Desrut et al., 2020; Huang et al., 2020; Zhang et al., 2020; Zhao et al., 2020
*SWEET16*	Glucose, fructose, sucrose	TP	Overexpression shows altered germination rate, growth phenotype, and stress tolerance	Blommel et al., 2004; Klemens et al., 2013; Guo et al., 2014; Walerowski et al., 2018; Sellami et al., 2019; Aubry et al., 2021
*SWEET17*	fructose	TP	fructose homeostasis regulation	Chardon et al., 2013; Guo et al., 2014; Veillet et al., 2017; Walerowski et al., 2018; Aubry et al., 2021

Substrates, subcellular localization, and physiological roles of Arabidopsis SWEET family members.

Abbreviations: 2-DOG, 2-deoxyglucose (glucose analog); PM, plasma membrane; *RPG1* or *2*, *RUPTURED POLLEN GRAIN 1* or *2*; *SAG29*, *SENESCENCE ASSOCIATED GENE 29*; TGN, *trans*-Golgi network; TP, tonoplast; *VEX1*, *VEGETATIVE CELL EXPRESSED 1*.

Given the comparatively high number and diverse chemical properties of amino acids (i.e. charge, polarity, aromaticity), the substrate recognition and transport mechanism of UmamiTs is likely more complex compared to that of SWEETs. While some prokaryotic DMTs, like YddG, seem to specifically transport aromatic amino acids, YdeD exports cysteine, asparagine, and glutamine, RhtA exports threonine and homoserine, and *Rickettsia prowazekii* Sam imports S-adenosylmethionine (Franke et al., 2003; Livshits et al., 2003; Tucker et al., 2003; Doroshenko et al., 2007; Tsuchiya et al., 2016). A similar preference can currently not be attributed to any UmamiTs, based on the admittedly limited data available. Phenylalanine—the sole aromatic amino acid included in UmamiT transport assays so far—was shown to be a substrate for UmamiT14, 24, and 25 (Besnard et al., 2016, 2018). However, the same transporters were also able to transport up to 13 additional proteinogenic amino acids and structurally related metabolites (Table 3, Supplemental Figure S2). Interestingly, UmamiT5/WAT1 (WALLS ARE THIN 1) facilitates vacuolar influx of indole-3-acetic acid, which is structurally similar to tryptophan (Ranocha et al., 2013). Phylogenetic evidence indicates that *At*UmamiT5 belongs to a distinct clade (clade V; Supplemental Figure S2) that contains UmamiT1, also in the tonoplast (Schmidt et al., 2007). It is tempting to speculate that these members all mediate auxin transport across the tonoplast. γ-aminobutyric acid was transported by UmamiT23-25 (Besnard et al., 2018), citrulline by UmamiT18/SIAR1 (SILIQUES ARE RED 1; Ladwig et al., 2012). Though initially thought to be plasma membrane transporters, some UmamiTs localized to the vacuole and/or the ER (Table 3; Supplemental Figure S2).

**Table 3:** UmamiTs in Arabidopsis

Gene Name (Alternative Name)	Substrate(s)	Locali-zation	Physiological Role	References
*UmamiT1*	N.D.	TP	N.D.	Schmidt et al., 2007
*UmamiT5* (*WAT1*)	auxin (IAA)	TP	Vacuolar auxin influx	Ranocha et al., 2010, 2013
*UmamiT11*	Gln	PM	Likely cellular efflux to support embryo growth	Müller et al., 2015
*UmamiT14*	Glu, Phe, Gln/Arg, Ala, Ser, Gly, Asn, Pro, Thr, Val, His, Ile, Leu, citrulline	PM	Likely cellular efflux to support embryo growth, phloem unloading in roots	Müller et al., 2015; Besnard et al., 2016
*UmamiT18* (*SIAR1*)	Asp, Gln/Arg, Ala, Asn, Thr, Val, His, Ile, Leu	PM	Phloem unloading in roots, apoplasmic release of amino acids in seeds	Ladwig et al., 2012; Besnard et al., 2016
*UmamiT23*	Gln/Arg, Glu, GABA, Asp, Thr	N.D.	N.D.	Besnard et al., 2018
*UmamiT24*	Gln/Arg, Ala, Glu, GABA, Phe, Val, Gly, Asp, Thr, Ser, Ile	TP	Involved in transient storage of amino acids	Besnard et al., 2018
*UmamiT25*	Gln/Ala, Glu, Leu, GABA, Phe, Val, Gly, Asp, Thr, Ser, Ile, Pro	PM	Amino acid export from the endosperm	Besnard et al., 2018
*UmamiT28*	Gln	PM	Likely cellular efflux to support embryo growth	Müller et al., 2015
*UmamiT29*	Gln	PM	Likely cellular efflux to support embryo growth	Müller et al., 2015
*UmamiT36* (*RTP1*)	N.D.	ER	N.D.	Pan et al., 2016

Substrates, subcellular localization, and physiological roles of Arabidopsis UmamiT family members.

Abbreviations: ER, endoplasmic reticulum; GABA, γ-aminobutyric acid; IAA: indole-3-acetic acid; *RTP1*, *RESISTANCE TO PHYTOPHTHORA PARASITICA 1*; *SIAR1*, *SILIQUES ARE RED 1*; TP, tonoplast; *WAT1,* *WALLS ARE THIN 1*.

## Transport mechanisms of SWEETs and UmamiTs

The study of sugar transport mechanism in plants started more than 40 years ago (Giaquinta, 1976). Since then, various sugar transporters from different species were characterized in heterologous expression systems (e.g. Riesmeier et al., 1992; Gahrtz et al., 1994; Sauer and Stolz, 1994; Carpaneto et al., 2005; Chen et al., 2012). SUTs, the first identified sucrose transporters, share common features with amino acid permeases (AAPs). Both SUTs and AAPs cotransport protons and sucrose (SUT1) or amino acids (AAPs) into cells at a stoichiometry of 1:1 (Fischer et al., 1995; Boorer et al., 1996a, 1996b). As proton symporters, their activity is determined by the proton motive force.

SWEETs are characterized by their ability to mediate bidirectional transport, their low-affinity for sugars, and pH-independency (Chen et al., 2012). In the absence of direct evidence, these characteristics are consistent with SWEETs functioning as uniporter, meaning that the concentration gradient of sugar determines whether flux is inward or outward. This is consistent with physiological observations, which support import in a few cases and efflux in many (see below).

Amino acids can occur as positively/negatively charged or neutral molecules. Although heterologous expression of UmamiTs revealed their bona fide ability to bidirectionally transport certain amino acids (Ladwig et al., 2012; Müller et al., 2015), physiological evidence is consistent with a uniport mechanism for UmamiTs. Thereby positively charged amino acids would show a tendency to be taken up into the cell, while negatively charged amino acids would rather exit the cell—even against a concentration gradient. Other transport mechanisms, such as a proton symport-coupled uptake (as used by AAPs) or proton antiport-mediated export of amino acids would be needed to allow the transport of amino acids against their (electro)chemical gradient. Based on the YddG crystal structure, a unique alternating-access transport mechanism was proposed, characterized by bending motions of transmembrane segments 3, 4, and 9 (Tsuchiya et al., 2016).

## Roles for SWEETs in phloem loading

The identification of SUT sucrose/H^+^ symporters and the demonstration that SUT1 homologs were essential for phloem loading in potato (*Solanum tuberosum*), tobacco (*Nicotiana tabacum*), Arabidopsis, and maize (*Zea mays*) implicated a yet unidentified mechanism for sucrose export for cells along the path from synthesis in the mesophyll to the sites of loading at the sieve element-companion cell complex (SECC; Riesmeier et al., 1992, 1993, 1994; Bürkle et al., 1998; Gottwald et al., 2000; Slewinski et al., 2009). Although ample evidence had been assembled for the existence of the sucrose/H^+^ symporters and their role in importing sucrose into the SECC, essentially nothing was known about proteins involved in the efflux of sucrose and their location in the leaf. Various quantitative studies on the distribution of plasmodesmata had implied the interface between the SECC and the adjacent phloem parenchyma (PP) inside the phloem as the apoplasmic transfer site.

### Arabidopsis

**Figure 3:**
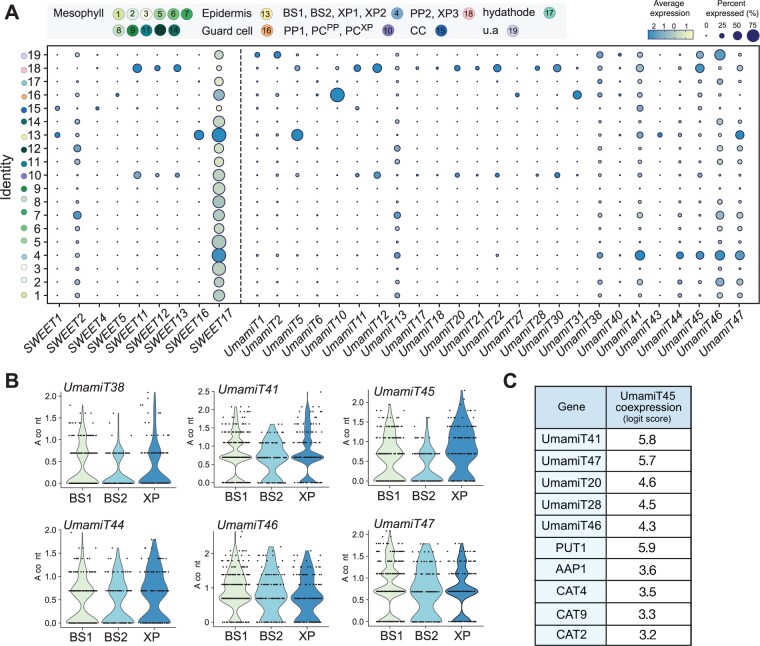
Cell type-specific transcript enrichment of *SWEET* and *UmamiT* family genes. A, Dot plot showing transcript enrichment of *SWEET* and *UmamiT* family genes across 19 clusters of the leaf scRNA-seq data (Kim et al., 2021). The diameter of the dot indicates the percentage of cells in the cluster in which transcripts for that gene were detected, while the color of each dot represents the average log-scaled expression of each gene across all cells within a given cluster. Cell types assigned to each cluster are indicated in the upper panel. Note that only *SWEET* and *UmamiT* family transcripts detected in the leaf scRNA-seq dataset were included in the plot. For detailed information about the dataset and description of the clusters/subclusters, refer to Kim et al. (2021). B, Violin plots illustrating the transcript enrichment of clade VI *UmamiT38*, *UmamiT41*, *UmamiT45*, *UmamiT44*, *UmamiT46*, and *UmamiT47* in the subclusters of cluster 4. C, *UmamiT45*-coexpressed genes related to amino acid transport. Coexpression data were obtained from the ATTED-II coexpression database (http://atted.jp). The logit score (MR, mutual rank; Obayashi et al., 2014) for the *UmamiT45*-coexpressed *UmamiTs*, AAP family protein *PUT1* (At1g31820), *AAP1* (At1g58360), cationic amino acid transporters *CAT4* (At3g03720), *CAT9* (At1g05940), and *CAT2* (At1g58030) is indicated. The logit score for *UmamiT45* was 14.2.

**Figure 4:**
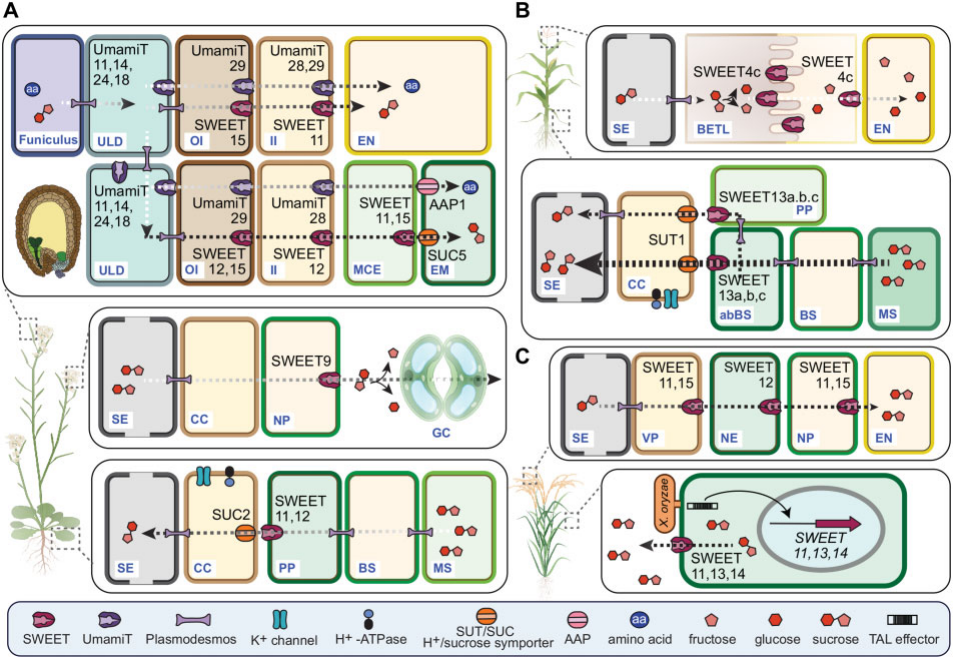
The role of SWEETs and UmamiTs in Arabidopsis, rice, and maize. A, The role of SWEETs and UmamiTs during seed filling (upper panel), nectar secretion (middle panel), and phloem loading in Arabidopsis (lower panel). Tissues of the seed (marked with distinct colors) are shown in the top left illustration. The same colors refer to tissues depicted in the panel. Note that the spatial distribution of SWEETs and UmamiTs changes dynamically during seed development. The schematic presented here illustrates early developmental stages (heart stage). B, The role of SWEETs in seed filling (upper panel) and phloem loading in maize (lower panel). C, The role of rice SWEETs in seed filling (upper panel) and pathogen growth (lower panel). Arrows indicate the direction of sugar or amino acid flow. OI, outer integument; II, inner integument; MCE, micropylar endosperm; EN, endosperm; EM, embryo; SE, sieve element; NP, nectary parenchyma; GC, guard cell; BS, bundle sheath; BETL, basal endosperm transfer layer; ^ab^BS, abaxial bundle sheath; VP, vascular parenchyma; NE, nucellar epidermis; NP, nucellar projection. Figure was created with Biorender.

SWEET11 and 12 were shown to be expressed in specific phloem cells, most likely the PP (Chen et al., 2012). Single-cell RNA-sequencing (scRNA-seq), in combination with confocal microscopy enabled unambiguous assignment of SWEET11 and SWEET12 to the PP (Kim et al., 2021; Figure 3A). The phenotype of T-DNA mutants was consistent with the role of these two SWEETs in sucrose efflux from PP (Chen et al., 2012). The resulting model for phloem loading assumes that sucrose produced by photosynthesis in mesophyll cells (MCs) is transported to PP through plasmodesmata. Sucrose is then exported into the apoplasm by SWEET11 and SWEET12. Sucrose is then taken up actively into the SECC by SUT1/SUC2, energized by H^+^-ATPases (Figure 4A). However, there are some caveats to this model—if apoplasmic transport functioned as the exclusive path, ablation of key members is expected to be lethal. However, *suc2* and *sweet11;12* double mutants are both viable and fertile (Srivastava et al., 2008; Chen et al., 2012). Therefore, it is likely that other transporters or other routes exist. Notably, mRNA levels of *SWEET13* was increased in *sweet11;12* mutants (Chen et al., 2012). The presence of *SWEET13* transcripts in the same cells as *SWEET11* and *12* indicates additive activities (Figure 3A; Kim et al., 2021). Likely, distinct routes coexist, possibly using plasmodesmata.

### Maize

Although the leaf morphology of C4 monocotyledous species with Kranz anatomy is distinct, maize uses a homolog of the Arabidopsis SUT1/SUC2—named *Zm*SUT1 for phloem loading as well (Slewinski et al., 2009). Phylogenetically, *Zm*SUT1 does not belong to the same dicot branch but fulfills the same function of supplying the SECC with sucrose. In maize, the closely related *SWEET13s* (*SWEET13a*, *b*, and *c*) are among the genes with the highest transcript levels in leaves (Emms et al., 2016; Bezrutczyk et al., 2018a). Knock-out mutants show symptoms of severe phloem loading defects. A combination of scRNA-seq, in situ hybridization, and translational GUS fusions indicates that all three *SWEET13*s are preferentially expressed in the two abaxial bundle sheath cells of the rank-2 intermediate veins (cells responsible for sucrose export from leaves; Fritz et al., 1983, 1989; Bezrutczyk et al., 2021). As *sut1* and *sweet13a;b;c* triple mutants are viable and fertile, maize also appears to use additional phloem loading pathways or compensate in yet unknown ways (Botha, 2013; Figure 4B).

### Rice

It was tempting to hypothesize that rice (*Oryza sativa*) homologs of *Zm*SUT1 and *Zm*SWEET13 would be key players for phloem loading in rice. However, neither mutants in *OsSUT1* nor in the closest *OsSWEET13* showed symptoms of phloem loading defects (Eom et al., 2012, 2019). Thus, despite the phylogenetic relationship among grasses, distinct phloem loading mechanisms seem to exist. Interestingly, another member of the SUT family, namely *Os*SUT2, might provide the driving force for phloem loading via symplasmic mechanisms (Eom et al., 2012). *Os*SUT2, as a vacuolar sucrose/H^+^ symporter, could be responsible for extremely high levels of sucrose in the cytosol of MCs, which could enable diffusion down a concentration gradient to sieve elements via plasmodesmata. Since sucrose concentration is estimated to reach almost 600 mM in rice (Hayashi and Chino, 1990), very higher sucrose concentrations would be needed for efficient translocation.

## Physiological roles of UmamiTs

Transcripts from most *UmamiT*s belonging to clade VI (Supplemental Figure S2) were broadly detected in cells from almost all cell types of the leaf, with an apparent preferential accumulation in cells from the bundle sheath/xylem cells, overlapping with transcripts from other amino acid transporters (Figure 3, A–C). More cell type-specific expression was observed for *AtUmamit5/WAT1* in epidermal cells, whereas transcripts of *AtUmamiT10*, *27*, and *31* were almost exclusive for guard cells (Figure 3A), pointing toward stomatal functions. As cell types in the leaf have distinct metabolic activities reflected by the differential transcript level of metabolic pathway genes (Kim et al., 2021), it will be interesting to assess the substrate specificity and the role of these UmamiTs in respect to the cell types where they are enriched (Box 1). More detailed analyses were performed using reporters for a number of UmamiT family members, yielding evidence for roles in phloem loading and seed filling.

Box 1 single-cell RNA-seq as an approach to identify additional transporters

Single-cell transcriptomics is a rapidly evolving field that enables profiling transcriptomes of individual cells derived from complex organs. This revolutionary technology was made possible by capturing individual cells and sequencing low amounts of RNA. A major goal of single-cell transcriptomic studies is to obtain transcriptome signatures of individual cells and cluster distinct cell types (or states) within complex tissues and associate these transcriptomic cellular states with the functional state of each cell type. scRNA-seq has been applied to different tissues of diverse plant species (Denyer et al., 2019; Jean-Baptiste et al., 2019; Ryu et al., 2019; Shulse et al., 2019; Zhang et al., 2019; Liu et al., 2020, 2020; Wendrich et al., 2020; Bezrutczyk et al., 2021; Kim et al., 2021). These studies identified the transcriptomes of major cell types or subtypes (or states) that were not previously well defined, for example, vascular cell types in different developmental states.

Recently, computational pipelines were established to characterize the metabolic heterogeneity with cell type resolution based on single-cell data (Xiao et al., 2019; Box 1, Figures A and B). Using these approaches, the activity of metabolic pathways can be assessed at the cell type level (Xiao et al., 2019; Kim et al., 2021).

Panel A shows a Uniform Manifold Approximation and Projection (UMAP) dimensional reduction projection of transcriptome profiles from 5,230 Arabidopsis leaf cells grouped into distinct clusters (Kim et al., 2021). Each dot represents an individual cell colored according to cell type. Panel B shows a UMAP plot filtered for metabolic genes (same dataset), demonstrating that clustering patterns are retained but shifted, intimating distinct expression patterns for metabolic genes (Kim et al., 2021). This approach may help identify transporters, as, depending on the substrate, transporters likely correlate with the respective metabolic pathways of that cell.
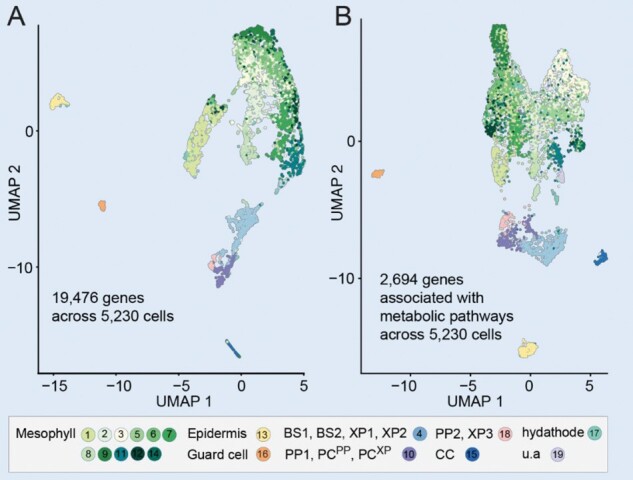

**Figure:** Landscape of metabolic gene expression in single leaf cells. A, UMAP plot of metabolic gene expression profiles of leaf cells from the Arabidopsis leaf scRNA-seq dataset (Kim et al., 2021). UMAP was used for visualization by reducing data to two-dimensions. Each dot indicates one cell; colors indicate cell type described in the legend (BS1, bundle sheath; BS2, bundle sheath cells enriched with photosynthetic processes; XP1 and XP2, xylem cells related with the bundle sheath; XP3, xylem cells enriched with vascular parenchyma markers; PCXP, procambium cells related to XP1; PCPP, procambium cells related to PP cells with transfer cell identity (PP1); CC, companion cells, u.a., unassigned). B, UMAP plot of metabolic gene expression profiles of same dataset as in (A).

## Roles of UmamiTs in phloem loading

Amino acids are the main transport forms of organic nitrogen in the phloem of most plants. We may therefore hypothesize to find similar pairs of transporters analogous to the SWEET-SUT pair for organic nitrogen. In accordance with a role in amino acid efflux from PP in Arabidopsis, transcripts of multiple *UmamiTs* were enriched in the same cells as transcripts from *SWEET11*, *12*, and *13.* Six out of seven PP-specific *UmamiT*s are coexpressed with each other and with *SWEET11* and *12* (Kim et al., 2021; Figure 3A). Similar to SWEET11 and 12, UmamiT18/SIAR1 is expressed both in the PP of leaves and in seeds (Ladwig et al., 2012; Kim et al., 2021). Mutants show reduced amino acid accumulation in seeds, possibly implicating UmamiT18 in transport processes in phloem loading as well as seed filling (Ladwig et al., 2012; Kim et al., 2021). These similarities to sucrose transport are striking, since also in this case multiple SWEETs contribute to phloem loading, and SWEETs have dual roles in phloem loading and seed filling. The large number of *UmamiT*s that are coexpressed in PP, may indicate that they are needed to maximize flux and cover the diverse set of substrates. Transcripts of *AAP2*, *AAP4*, and *AAP5* amino acid H^+^/symporters are enriched in companion cells (CCs), and may thus play analogous roles for importing amino acids as SUTs do for sucrose (Kim et al., 2021). Notably, the amino acid metabolism in PP and CC is very different, indicating that metabolic activities shape the amino acid composition of the phloem sap (Kim et al., 2021; Box 1). Further characterization of UmamiTs and cell specific metabolism will be useful to understand the regulation of amino acid allocation.

## Roles for SWEETs and UmamiTs in phloem unloading

The partitioning of sucrose and amino acid is strongly dependent on phloem loading in the source regions and unloading in the sink regions of the plant. Several SWEETs and UmamiTs from various species are known to be expressed in different sink tissues (Figure 4; e.g. Kryvoruchko et al., 2016; Zhen et al., 2018; Jeena et al., 2019; Wang et al., 2019; Ren et al., 2021). Impaired activity of SWEETs or UmamiTs causes defects in basic physiological processes reflecting their broad role. Additional roles of SWEETs and UmamiTs in pathogen susceptibility have also been reported in numerous studies and are summarized in Box 2 (Buell et al., 2003; van Damme et al., 2009; Chen et al., 2010; Ranocha et al., 2010; Smeekens et al., 2010; Denancé et al., 2013; Zeier, 2013; Hahn et al., 2014; Schwelm et al., 2015; Struck, 2015; Bezrutczyk et al., 2018b; Besnard et al., 2021; Prior et al., 2021).

Box 2 SWEETs and UmamiTs: roles in pathogen susceptibility

Upon identification of the SWEETs, their involvement in pathogen susceptibility was promptly reported, that is, upregulation of *Os*SWEETs during *Xanthomonas oryzae pv oryzae* (*Xoo*) infection*.* Later, the bacterial blight resistance-conferring locus *Xa13* was shown to correspond to *Os*SWEET11 (originally called *Os*8N3), and *xa25* and *xa41* to *Os*SWEET13 and *Os*SWEET14, respectively. Noteworthy, only sucrose-transporting clade III SWEETs function as susceptibility genes. Induction of these *SWEETs* occurs by binding to their promoters of *Xoo*-secreted TAL (transcriptional activator-like proteins) effectors (Figure 4C). Interestingly, North American *Xoo* isolates lack TAL effectors and are weak virulence-inducing pathogens compared to African/Asian strains. Therefore, the ability to induce SWEETs was likely crucial for turning *Xoo* into a “super pathogen.” SWEETs from several plant species are induced by diverse pathogens, including biotrophic bacteria, oomycetes, and fungi (Chen et al., 2010; Smeekens et al., 2010; Bezrutczyk et al., 2018b). As the genomes of various pathogens lack TAL effector homologs (Buell et al., 2003; Hahn et al., 2014; Schwelm et al., 2015), these pathogens possibly use alternative *SWEET* induction mechanisms. In Arabidopsis, *Pseudomonas syringae (Pst* DC3000) presumably activates a *bZIP* transcription factor that eventually induces *SWEETs* and *UmamiTs* (Prior et al., 2021). Pathogens likely activate *SWEETs* to gain access to carbon skeletons, energy, and nutrients for efficient reproduction.

Changes in the free amino acid pool composition/homeostasis affect plant defense responses (van Damme et al., 2009; Zeier, 2013) and pathogen nutrition (Struck, 2015). In addition, the expression of genes coding for amino acid transporters was altered upon pathogen infection (Pratelli and Pilot, 2014).

Implication of UmamiTs in pathogen susceptibility was shown repeatedly and is mediated by increased salicylic acid (SA) levels. For instance, auxin-transporting *At*UmamiT5/WAT1 is required for secondary cell-wall deposition (Ranocha et al., 2010), and *wat1* mutants conferred broad-spectrum resistances against vascular pathogens, likely due to reduced root auxin levels (Denancé et al., 2013).

Likewise, *AtUmamiT36/RTP1* (*RESISTANCE TO PHYTOPHTHORA PARASITICA 1*) mutants displayed increased *Pst* DC3000 and *G. cichoracearum* resistance (Pan et al., 2016). Enhanced pathogen resistance upon overexpression of a *Mt*N21-like amino acid transporter was shown for *UmamiT14*.

Plants overexpressing *UmamiT14* displayed increased SA-levels and enhanced resistance toward the biotrophic *Hyaloperonospora arabidopsidis*, likely due to a constitutive immune response (Besnard et al., 2021). Together, these results suggest that misregulation (i.e. up-/downregulation) of *UmamiTs* and subsequent altered amino acid accumulation/composition can trigger enhanced pathogen resistance, albeit without clear correlations to changes in specific amino acids.

### Roles for SWEETs in seed filling

Growth and development of the embryo depends on adequate supply with photoassimilates from maternal tissues. The unfertilized ovule is symplasmically isolated from the maternal tissues before fertilization. Post fertilization, a drastic increase of plasmodesmata can be observed between the terminal sieve element and neighboring cells at the chalazal region (the seed nutrient unloading zone) forming a symplasmically connected unloading domain (ULD). Along the unloading path, the unloading zone and the integuments, the layers between outer and inner integuments, and endosperm and the embryo are symplasmically isolated (Thorne, 1985; Stadler et al., 2005; Werner et al., 2011). Consequently, sugars and amino acids must be exported from one cell into the apoplasm before then can be reimported in the adjacent cell.

One of the most elegant systems for studying metabolite efflux is the “empty seed technique” established in legumes (Wolswinkel and Ammerlaan, 1983; Thorne, 1985; Fieuw and Patrick, 1993; Walker et al., 2000). In this technique, the embryo is surgically removed and the seed coat is filled with solutions known to influence assimilate transport. By assaying the contents of the solution, it is possible to study transport processes involved in the release of assimilates to the developing seed. The studies revealed that sucrose efflux from the seed coat occurs, in part, by sucrose/H^+^ antiport (in part mediated by SUT; Baud et al., 2005; Zhang et al., 2007), as well as by proton gradient-independent mechanisms (Walker et al., 1995; De Jong et al., 1996). Consistent with their function as uniporters, SWEET members could contribute to the proton-independent efflux from the seed coat. The cell type specificity of SWEET4, 11, 12, 15 is developmentally regulated and each of them likely contributed to sucrose transfer across the different symplasmic barriers (Chen et al., 2015c; Lu et al., 2020). Triple *sweet11;12;15* mutants accumulated starch in the seed coat and showed severe defects in seed development, implicating important roles in sucrose efflux at distinct steps in seed filling (Chen et al., 2015c; Figure 4A). However, as *sweet11;12;15* mutants were viable, additional transport mechanisms are likely to exist. It is likely that a yet to be identified sucrose/H^+^ antiporters may be responsible for this function (Fieuw and Patrick, 1993; Walker et al., 1995).

The evidence for SWEETs in feeding tissues in the developing seed is not limited to Arabidopsis, but also exists in crops. In rice, *Os*SWEET11 and 15 are essential for transporting sugar through distinct apoplasmic pathways (Figure 4C; Ma et al., 2017; Yang et al., 2018). In maize, *ZmSWEET4c*, *ZmSWEET11*, and *ZmSWEET15b* were found to be localized at different stages of seed development (Sekhon et al., 2011; Li et al., 2014; Sosso et al., 2015). *Zm*SWEET4c is likely involved in translocating cell wall invertase-derived hexoses in and/or across the basal endosperm transfer layer (BETL), a cell layer of endosperm characterized by cell wall invaginations that amplify the plasma membrane surface area (Sosso et al., 2015; Figure 4B). Interestingly, ZmSWEET4c may be a target of domestication that was likely recruited by farmers and breeders who selected for large grains. Although substantial progress has been made, the full path of sucrose in none of the species has been unraveled.

### Roles for UmamiTs in amino acid supply to seeds

The transport of amino acids and sucrose shares commonalities since both processes must undergo similar symplasmic and apoplasmic steps. Uptake of amino acids into the embryo has been shown to occur via H^+^/amino acid symporters such as the AAPs, while efflux processes are mediated by proton gradient-independent, transporter-mediated mechanisms (Lanfermeijer et al., 1990; de Jong et al., 1997; Tegeder et al., 2000; Sanders et al., 2009; Zhang et al., 2015; Karmann et al., 2018). Analogous to the roles of several SWEETs, multiple UmamiTs are implied in proton-independent efflux of amino acids to supply the developing seed (Karmann et al., 2018; Figure 4A). Interestingly, several UmamiTs localize to the ULD where symplasmic transport through plasmodesmata is considered as the dominant route (Stadler et al., 2005). In the plasma membrane of the ULD, UmamiT11, 14, 18, and 24 have been implicated in the export of amino acid to the developing embryo (Figure 4A;Table 3). In early seed development, UmamiT11 and 14 are present in cells at the end of the funicular vasculature, which are adjacent to the protoxylem, CCs, and sieve elements. In accordance with a disrupted export of amino acids from the chalazal zone, seeds of *umamit11* and *14* single mutants were smaller (Müller et al., 2015). Only at later stages (torpedo stage), UmamiT28 was detected in the cellularizing endosperm and the endothelium layer of the inner integuments (Müller et al., 2015). Up to the late torpedo stage of embryo development, UmamiT29 was found in the middle layer of the inner integument, followed by a shift in localization to the inner layer of the outer integuments in later developmental stages (Müller et al., 2015). Mutants of either *UmamiT28* or *29* produced smaller seeds with a trend to accumulate amino acids (Müller et al., 2015). Contrarily to the tonoplast-localized UmamiT24, which might be involved in temporary amino acid storage, the plasma membrane-localized UmamiT25 presumably mediates amino acid export from the endosperm (Besnard et al., 2018). Mutations in either gene resulted in reduced seed amino acid content, likely due to reduced uptake (Besnard et al., 2018). Taken together, the developmentally controlled differential expression of UmamiTs across several symplasmic seed tissues is suggestive of their roles in transfer of amino acid export from maternal to filial tissues. The relatively high number and overlapping expression patterns of *UmamiTs* involved in amino acid transport in seeds point toward redundant functions for at least some of the proteins (Müller et al., 2015). However, the full path of amino acid translocation remains to be unraveled.

#### Roles for SWEETs in pollen nutrition

Pollen germination and tube growth initially rely on nutrient storage in the pollen grain. Pollen grains, pollen tubes, and the anther tapetum are sink tissues that are symplasmically isolated, requiring an unloading pathway through the apoplasmic space. In Arabidopsis, *At*SWEET8 and *At*SWEET13, also known as *RUPTURED POLLEN GRAIN* (*RPG*) *1* and *2*, respectively, were suggested to function in the efflux of sugar in the tapetum and microspores for pollen cell wall synthesis (Guan et al., 2008; Sun et al., 2013; Kanno et al., 2016). Mutations in *SWEET8* and *SWEET13* resulted in pollen cell wall defects and reduced male fertility. *At*SWEET13 could partially rescue the defective pollen phenotype of *atsweet8*, suggesting functional redundancy (Sun et al., 2013). In coniferous Wilson’s spruce (*Picea wilsonii*), *Pw*SWEET1 was implied in supplying glucose for proper pollen germination and pollen tube growth (Zhou et al., 2020). In rice, *Os*SWEET11 has been implicated in the export of sugars during pollen development as *OsSWEET11*-silenced plants showed low fertility and pollen viability (Yang et al., 2006; Eom et al., 2015).

#### Roles for SWEETs in nectar secretion

To attract and reward pollinators, plants secrete nectar—a sugar-rich solution, which contains volatile compounds produced in the nectary. The mechanism for nectar secretion was reported through a study using Arabidopsis, turnip (*Brassica rapa*), and coyote tobacco (*Nicotiana attenuate*; Lin et al., 2014). Arabidopsis *SWEET9* is highly expressed in the nectary and similar as its homologs in *N. attenuata* and *B. rapa* show sucrose uniport activity. Loss of *AtSWEET9* resulted impaired nectar secretion. The current model proposes that sucrose synthesized in the nectary parenchyma cells is secreted via *At*SWEET9 into the apoplasm and hydrolyzed by cell wall invertases, which cause an osmotic gradient to sustain water secretion (Figure 4A). However, as secreted nectars require a fast and active secretion, it likely cannot solely be mediated by uniport. How *At*SWEET9, as a uniporter, can secrete sugar to high levels is still a conundrum and whether other mechanisms are involved in the process remains to be elucidated. In petunia, NEC1, the homolog of SWEET9, is highly expressed in nectaries and likely plays a similar role (Ge et al., 2000).

#### SWEETs in vacuolar transport

The vacuole occupies more than 80% of the plant cell volume and is separated from the cytosol by a semi-permeable membrane, the tonoplast. The vacuole is the primary compartment for maintaining cellular homeostasis, turgor pressure, detoxification, and importantly, storage of sugars. After the identification of the first tonoplast-localized monosaccharide transporter (Wormit et al., 2006), multiple vacuolar transporters mediating transport of sugars by facilitated diffusion and active transport have been described (e.g. Aluri and Buttner, 2007; Eom et al., 2011; Payyavula et al., 2011; Poschet et al., 2011; Schulz et al., 2011; Pommerrenig et al., 2018). Clade IV SWEETs have been shown to be responsible for the efflux of fructose, glucose, and sucrose from the vacuole (Jeena et al., 2019). *At*SWEET17, the first characterized vacuolar fructose transporter, was implied in determining leaf fructose content under normal and stress conditions (Chardon et al., 2013). *AtSWEET17* and its close paralog *AtSWEET16* were also shown to be highly expressed in the root vacuoles. Mutations and overexpression of *SWEET16* and *SWEET17* resulted in various growth phenotypes under normal and abiotic stress conditions, reflecting the vital role of vacuolar SWEET-mediated sugar efflux in the development and stress tolerance of plants (Chardon et al., 2013; Klemens et al., 2013; Guo et al., 2014). *At*SWEET2, another tonoplast-enriched clade I SWEET, has been hypothesized to prevent sugar loss from roots (Chen et al., 2015a). *AtSWEET2* was also shown to be induced during *Pythium* infection. As *sweet2* mutants were susceptible to the oomycete, it has been predicted that *At*SWEET2 modulates sugar secretion to limit carbon loss to the rhizosphere. A detailed description of the role of SWEETs is presented in Table 2.

## Nutrition of symbiota and microbiota

The observation that SWEETs and UmamiTs were originally identified as nodulins may indicate possible roles in symbiosis (Box 3). Besides the highly evolved symbiotic system in legumes, many plants are colonized by mycorrhiza and rhizobials, and all plants interact closely with endo- and ectophytic microbiota. Notably, plants are thought to secrete 15%–40% of their photosynthate into the soil, presumably for feeding root-colonizing microbiota (Lynch and Whipps, 1990). Although the composition of exudates is influenced by various factors, primary metabolites including sugars, amino acids, and organic acids, are secreted in larger quantities than secondary metabolites (Badri et al., 2008). For instance, in maize, sugars constitute 64% of the root exudate, whereas amino acids and low molecular weight organic acids (LMWOAs) represent 22% and 14%, respectively (Hütsch et al., 2002).

Box 3 Symbiosis as a basis for microbiota establishment

The “symbiosis cascade effect” hypothesis describes how plant symbiosis establishment influences and subsequently drives the assembly plant root microbiota (Uroz et al., 2019). The establishment of symbiosis affects intra- and intercellular communication, transcriptional reprogramming, rerouting of metabolite signaling pathways, hence, root exudate composition and root architecture. In turn, this influences the establishment and/or modifies the microbial community structure (Uroz et al., 2019).

Root AMF and nitrogen-fixing/nodulation symbiosis affects the root microbiome. The root microbial community structure of *L. japonicus* mutants impaired in nodulation or symbiosis is similar among diverse mutants but distinct from that of the wild-type. The altered community structure was retained even in nitrogen-supplemented soil where nodulation is prevented in wild-type (Zgadzaj et al., 2016). Similarly, *M. truncatula* mutants impaired in nodulation and/or AMF symbioses assemble more similar root bacterial communities compared to wild-type (Offre et al., 2007). AMF and nodulation symbiosis play important roles in structuring the root fungal and bacterial communities, and disruption of symbiosis causes major shifts in bacterial and fungal assembly (Thiergart et al., 2019; Wang et al., 2020).

Numerous SWEETs across different plant families were shown to be induced during AMF and rhizobial symbiosis (Fiorilli et al., 2015; Manck-Götzenberger and Requena, 2016), indicating an evolutionarily conserved role for some SWEETs in symbiosis. Although the exact role of SWEETs in microbiotal sugar feeding remains yet unclear, their roles in AMF and rhizobia symbiosis are a compelling basis to speculate about a role for sugar transporters in shaping the root microbiota. It should be noted that while evidence for symbioses as a basis for root microbiota establishment exists, there are differences between symbiosis and root microbiota formation (Sasse et al., 2018).

### Sugars: *SWEETs*

**Figure 5:**
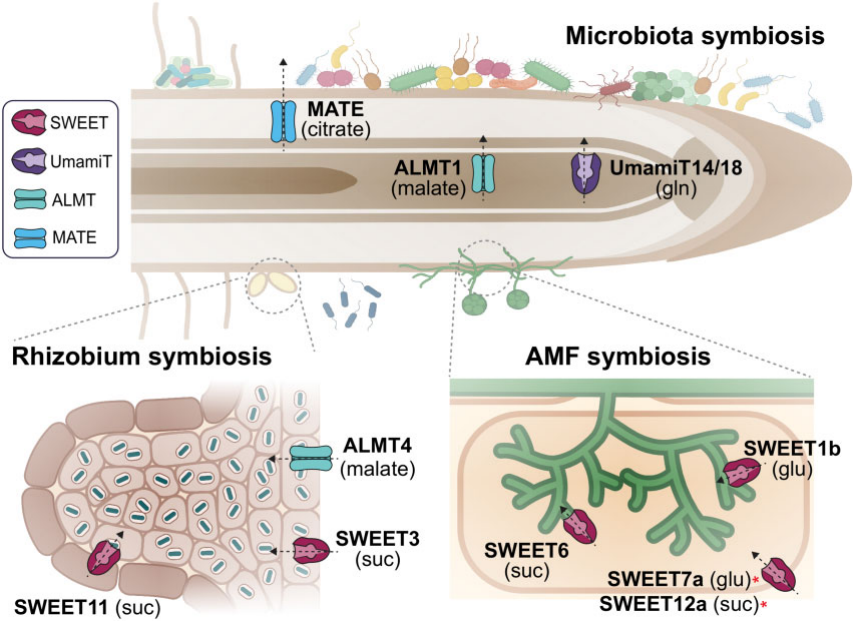
Transporters with potential roles in root metabolite efflux for feeding microbiota and symbiota. Microbes actively recruited to the proximities of the root surface (rhizosphere) or colonizing the internal root tissues (endosphere) constitute the root microbiota. Malate secretion by ALMT1 during pathogen challenge recruits *B. subtilis* for induced systemic resistance. ALMT1 also mediates the transport of malate into rhizobia-symbiosomes. Microbial-derived products trigger increased amino acid efflux in the roots, likely via UmamiTs. Sugar exported via SWEETs to arbuscule containing cells (AMF symbiosis) or nodules (rhizobia symbiosis) serves to maintain favorable growth conditions for symbiosis. MATE transporters are involved in the efflux of citrate, which can be metabolized by microbes. AMF and rhizobacteria symbioses trigger the symbiosis cascade effect that could be a basis for the establishment of mutualistic interactions in the root. Substrates for the corresponding transport proteins are indicated (gln, glutamine; glu, glucose, suc, sucrose). Asterisks indicate putative substrates based on cross-reference to homologs. Figure was created with Biorender.

Plants grown in a rhizospheric microbial culture showed significantly less sugar reuptake compared to cultures in sterile conditions (Kuzyakov and Jones, 2006). Considering the broad roles of SWEETs in sugar secretion, it is conceivable that they also play a role in sugar efflux from roots. Unsurprisingly, arbuscular mycorrhizal fungus (AMF) and nitrogen-fixing rhizobia induce SWEETs across different plant families (Fiorilli et al., 2015; Manck-Götzenberger and Requena, 2016). Studies in potato roots revealed that colonization by the AMF *Rhizophagus irregularis* affected steady state mRNA levels of 22 of 35 *StSWEETs. StSWEET2c, StSWEET7a*, and *StSWEET12a* showed the highest induction in arbuscule-containing cells (Manck-Götzenberger and Requena, 2016). Transcriptome profiles from AMF-colonized soybean (*Glycine max*) revealed induction of *GmSWEET6* and *GmSWEET15* in arbuscule-containing cells (Manck-Götzenberger and Requena, 2016). In Medicago, the glucose transporter *MtSWEET1b/SWEET1.2* was induced in arbuscule-containing cells where it localized to peri-arbuscular membranes, presumably to facilitate nutrient exchange between host plant and AMF during symbiosis (An et al., 2019). SWEETs have also been implicated in legume-rhizobium symbiosis. In lotus (*Lotus japonicus*), *SWEET3* is preferentially expressed in nodules infected by *Mesorhizobium loti*, indicating a role in sugar transfer toward rhizobia (Pini et al., 2017; SugiyaMa et al., 2017). *LjSWEET3* was induced also by AMF, hinting at common regulatory mechanisms for AMF and rhizobial symbiosis, that could influence the assembly of root microbiota (Zgadzaj et al., 2016; SugiyaMa et al., 2017; Uroz et al., 2019). In *Sinorhizobium meliloti*-infected Medicago, *Mt*SWEET11 translocated from plasma membranes to transcellular infection threads and symbiosomes (Table 4; Figure 5) presumably mediating efflux of sugar into the symbiosomes (Kryvoruchko et al., 2016). A summary of the role of SWEETs in symbiosis and microbiota-feeding is presented in Table 4 and Figure 5.

**Table 4:** Roles of root exudates and their efflux transporters in plant root–microbiota interactions

Role in Plant–Microbe Interactions				Reference(s)
Root Exudate		Attributed Transporter (Substrate)		
Sugar	AMF and rhizobia symbiosis mutants are incapable of assembling normal root and rhizosphere microbiota	*Gm*SWEET6, *Mt*SWEET1b (glu), *St*SWEET2c, *St*SWEET7a, *St*SWEET12a	Localized in arbuscules-containing cells, indicated to function in supplying sugar to AMFs	Manck-Götzenberger and Requena, 2016; An et al., 2019; Zhao et al., 2019
		*Lj*SWEET3, *Mt*SWEET11 (suc)	Induced in nodules, transfection threads, and symbiosomes induced by nitrogen-fixing and non-nitrogen fixing rhizobia	Offre et al., 2007; Zgadzaj et al., 2016; SugiyaMa et al., 2017; Thiergart et al., 2019; Wang et al., 2020

Amino acid	Microbial-derived products increase amino acids efflux from roots of plants grown under hydroponic conditions	*At*UmamiT14, *At*UmamiT18 (gln)	*umamit14* and *umamit18* show decreased amino acid export from roots	Besnard et al., 2016

LMWOAs	Exogenous application of LMWOAs present in rhizospheric exudates result in selection for growth-promoting taxa, and stimulation of soil microbial activities	*At*ALMT1 (malate)	Overexpression of *AtALMT1* and induction by MAMPs recruits beneficial rhizobacteria that induce plant immunity	Rudrappa et al., 2008; Lakshmanan et al., 2012; Kobayashi et al., 2013; Macias-Benitez et al., 2020
		*Lj*ALMT4 (malate)	Specifically expressed in nodule vasculature bundle, and involved in bidirectional transport of malate in nodules	Takanashi, 2016
		*Ta*ALMT1	*Taalmt1* assembles differentially enriched root bacterial OTUs compared to wild-type	Mahoney, 2017
	Citrate-supplemented soil causes decrease in the richness and diversity of the bacterial communities	*Mt*MATE67 (citrate)	Induced by *S. meliloti* and localized to nodules and symbiosomes. Transports citrate to increase Fe(III) availability for rhizobia	Kryvoruchko et al., 2018
		*Lj*MATE1 (citrate)	Induced by rhizobia and AMF. Supports nodule function by providing citrate iron translocation to nodules	Takanashi et al., 2013; Handa et al., 2015

Others		*At*PDR2	Increased phenolic compounds and reduced sugar content in root exudates of *atpdr2* increases relative abundance of beneficial bacterial OTUs	Badri et al., 2008;, 2009
	N.D.	*Pa*PDR1 (strigolactones)	Root exudates of mutants show reduced AMF hyphal branching and promotes parasitic seed germination. *A. thaliana* overexpressing *PaPDR1* increased secretion of synthetic strigolactone analog	Kretzschmar et al., 2012; Sasse et al., 2015; De Cuyper and Goormachtig, 2017
	N.D.	*At*PDR8 (scopoletin), *At*PDR9 (coumarin)	*Atpdr9* mutants assemble different root-associated microbiota compared to wild-type in Fe-limiting conditions. *atpdr9* is incapable of microbiota-mediated plant growth rescue	Fourcroy et al., 2016; Voges et al., 2019; Harbort et al., 2020
	Diterpenes: Diterpenoid-deficient maize mutant shows altered rhizospheric microbiota, largely attributed to deficiency of diterpene in roots	*Nt*PDR1	*NtPDR1* overexpressor indicated a role for *NtPDR1* in efflux of diterpenes from roots in response to biotic triggers	Crouzet et al., 2013; Murphy et al., 2021
	Triterpenes: Triterpene biosynthesis mutants/analysis and exogenous triterpene treatment indicated roles in regulating bacterial growth	N.D.	N.D.	Huang et al., 2019
	Isoflavonoid: Essential for plant-rhizobium symbiosis. Simulated exudation of the most abundant rhizobia-inducing flavonoids shows interaction with diverse soil bacteria	*Mt*ABCG10 (isoflavonoid)	Fungal elicitor treatment on RNAi silenced-*MtABCG10* shows increase in isoflavonoids in root exudates. Increased susceptibility to pathogenic fungi in mutants.	Subramanian et al., 2007; Banasiak et al., 2013; Poole et al., 2018

Abbreviations: ABCG, ATP-binding cassette transporter G; gln, glutamine; glu, glucose; MAMP, microbe-associated molecular pattern; OTU, operational taxonomic unit; PDR, pleiotropic drug resistance; suc, sucrose.

### Amino acids: UmamiTs and GDU1

Dyshomeostasis in free amino acid pool impacts the defense response of plants, which in turn, could affect symbiosis (van Damme et al., 2009; Zeier, 2013; Pratelli and Pilot, 2014; Struck, 2015; Pan et al., 2016; Besnard et al., 2021; Box 2). In sterile conditions, amino acid reuptake into roots was higher compared to exudation (Phillips et al., 2004). Addition of microbial products significantly increased net amino acid exudation, likely due to effects on the relative activity of amino acid secretion and passive uptake by root microbes (Phillips et al., 2004). Mutants of *AtUmamiT14* and *AtUmamiT18* transferred lower amino acid amounts from roots to media, implicating the two UmamiTs in amino acid secretion toward the rhizosphere (Besnard et al., 2016). *GLUTAMINE DUMPER 1* (GDU1), which is mainly expressed in the root vasculature is also postulated to play a role in amino acid secretion from roots as overexpressors efflux elevated amino acid amounts into media (Pratelli et al., 2010). The molecular function of GDU1, a single membrane spanning protein, is yet to be revealed. The role of UmamiTs and GDUs in amino acid exudation and reuptake in nonsterile soil-grown plants will be an important research target.

### LMWOAs (malate and citrate): ALMTs and multidrug and toxic compound extrusion transporters

Under certain stress conditions and in the presence of soil microbes increased exudation of LMWOAs (mainly citrate and malate) has been observed (Boldt-Burisch et al., 2019). Interestingly, structure and activity of soil bacterial communities changed more dramatically upon treatment with LMWOA, compared to sugar treatment (Shi et al., 2011; Macias-Benitez et al., 2020). Although we do not understand which transporters are involved in LMWOA efflux for nutrition of microbiota, candidate transporters for malate and citrate come from three transporters superfamilies (Table 1). Malate efflux at the plasma membrane and import into vacuoles is mediated by aluminum-activated malate transporters (ALMT) and tonoplast dicarboxylate transporters (TDT), respectively (Igamberdiev and Eprintsev, 2016; Frei et al., 2018). The first *ALMT* was isolated from aluminum-tolerant wheat (*Triticum aestivum*), followed by identification of a functional ortholog in Arabidopsis*, AtALMT1* (Sasaki et al., 2004; Hoekenga et al., 2006). Characterization of various ALMTs unveiled roles in a wide range of physiological processes, including plant–microbe interactions (Piñeros et al., 2008; Meyer et al., 2011; De Angeli et al., 2013; Ramesh et al., 2015). *Pseudomonas* infection, and in particular, bacterial elicitors and jasmonic acid signaling induce *AtALMT1* in the absence of a low pH or an aluminum-rich environment, leading to root secretion of malate, thereby promoting colonization by beneficial rhizobacteria and conferring systemic immunity against subsequent pathogen infection (Table 4; Figure 5; Rudrappa et al., 2008; Lakshmanan et al., 2012; Berendsen et al., 2018). In wheat, lines carrying either wild-type or mutant *TaALMT1* alleles showed differences in host-associated bacterial taxa, reminiscent of that caused by symbiosis disruptions (Thiergart et al., 2019; Wang et al., 2020). Whether such differences are attributed to malate secretion have not yet been tested (Mahoney et al., 2017). The malate efflux transporter *Lj*ALMT4 is highly expressed in *M. loti*-infected nodule junctions of young nodules and in vascular bundles of mature nodules (Figure 5). Interestingly, *Lj*ALMT4-mediated malate efflux was independent of aluminum ions (Takanashi et al., 2016).

The efflux of citrate is mediated by the multidrug and toxic compound extrusion (MATE) transporters, which were originally identified as energy-dependent efflux transporters in bacteria that confer drug resistance (Morita et al., 1998). Plant genomes carry a large number of MATE orthologs, also known as DETOXIFICATION (DTX) proteins, for example, 58 MATE members in Arabidopsis (Hvorup et al., 2003). Citrate-transporting MATEs were identified in barley (*Hordeum vulgare Al-ACTIVATED CITRATE TRANSPORTER 1, Hv*AACT1), sorghum (*Sorghum vulgare*, *Sb*MATE), Arabidopsis (*At*MATE), rice (*FERRIC REDUCTASE DEFECTIVE LIKE 1 and 4, Os*FRDL1 and 4), rice bean (*Vigna umbellate*, *Vu*MATE1, *Vu*MATE2), and maize (*Zm*MATE1; Furukawa et al., 2007; Magalhaes et al., 2007; Yokosho et al., 2009; Maron et al., 2010; Yokosho et al., 2011; Liu et al., 2018). Although their direct involvement in microbe feeding requires more research, the ability of MATE transporters to efflux citrate, which can be metabolized by microbes, might imply such roles (Figure 5;Furukawa et al., 2007; Baetz and Martinoia, 2014). The iron-activated plasma membrane citrate efflux transporter *Mt*MATE67 is induced by *S. meliloti* inoculation. *Mt*MATE67 localizes to plasma membranes of symbiosomes, also known as the peribacteriod membrane, where it primarily transports citrate into the symbiosome to increase the availability of Fe (III) for rhizobia (Kryvoruchko et al., 2018). Similarly, the nodule-specific citrate transporter *Lj*MATE1 supports nodule function by providing citrate for iron translocation to the nodule infection zone (Takanashi et al., 2013). Interestingly, *LjMATE1* is also upregulated during AMF *R. irregularis* infection (Handa et al., 2015). Table 4 and Figure 5 summarize the current state for ALMTs, MATE, and other organic transporters (not mentioned in text, but reviewed in Sasse et al., 2018; Stassen et al., 2021) in plant–microbe symbiosis.

## Concluding remarks and future perspectives

The pressure-flow hypothesis (Münch, 1930) is still the most widely accepted mechanism for long-distance phloem transport. However, despite many contributions since then, our current understanding of assimilate allocation is still limited. The phloem sap contains many hundreds of metabolites (Fiehn, 2003), and for most we have no clue how they are transported (see “Outstanding questions”). For instance, malate is an important constituent of the phloem sap which affects nitrate uptake by roots (Touraine et al., 1992). However, a bona fide malate transporter that effluxes malate from the PP or imports malate by proton symport into the SECC remains elusive. It is conceivable that some members of the dicarboxylate transporter family are involved in malate secretion from the PP (Taniguchi et al., 2002). However, selecting candidates for transport assays and verifying their physiological roles is challenging. As substrates which enter and leave the cells are largely dependent on the distribution and function of plasmodesmata and transmembrane transporters, transcriptomic profiles at the single-cell level serves as a reliable map for selecting transporter candidates for a wide range of metabolites, hormones, and even ions (Box 1; e.g. Denyer et al., 2019; Jean-Baptiste et al., 2019; Ryu et al., 2019; Shulse et al., 2019; Xiao et al., 2019; Zhang et al., 2019; Liu et al., 2020; Wendrich et al., 2020; Bezrutczyk et al., 2021; Kim et al., 2021). Single-cell analysis may also serve as tool to dissect the integration between plants and the pathogenic (or commensal microorganisms). Several SWEETs in rice have been implicated in disease susceptibility to *Xanthomonas oryzae* pv. *oryzae* (Chen et al., 2010; Smeekens et al., 2010; Bezrutczyk et al., 2018a,b; Box 2), yet our current knowledge on other efflux transporters that may function as susceptibility factors is limited. Although our current technology is limited in capturing eukaryotic RNAs with polyA tails, development for simultaneously capturing eukaryotic and prokaryotic transcripts at once, and performing a combined single-cell and spatial transcriptomics, will provide insight on host cell–pathogen interaction at the cell type and spatial resolution. Importantly, the development of genetically encoded biosensors targeted to various cellular compartments will empower dissection of the mechanisms for distribution and fluxes of different nutrients. We expect that these technologies will allow us to better understand symbiosis establishment, plant–pathogen interaction, and enable us to systematically engineer nutrient flux in plants to increase crop yield in the future.

## Supplemental data

The following materials are available in the online version of this article.

**Supplemental Figure S1**. Phylogenetic analysis of SWEET family proteins of Arabidopsis (*At*), *Oryza sativa* (*Os*, rice), and *Zea mays* (*Zm*, maize).

**Supplemental Figure S2**. The phylogeny of 46 members of the *A. thaliana* UmamiT family.

**Supplemental Table S1**. Gene IDs of SWEETs used for phylogenetic trees.

**Supplemental Table S2**. Gene IDs of UmamiTs used for phylogenetic trees.

**Outstanding questions**

- When/how do sugars/amino acids cross plasmodesmata?
- How are symplasmic and apoplasmic pathways coordinated?
- How is the efflux from one cell coordinated with capacity for uptake by adjacent cells?
- How is the demand-supply of sugars/amino acids in sink-source tissues coordinated?
- How are SWEETs/UmamiTs dynamically regulated during development/upon environmental cues?
- How are import and export processes coordinated during seed filling? By which transporters—especially the unloading of sucrose/amino acids in the unloading domain? What is the step-by-step path of sucrose/amino acids in model and crop plants?
- Do SWEETs/efflux transporters play roles in pathogen susceptibility to other diseases?
- Are there clade-specific UmamiT substrate and/or localization patterns?
- How is malate loaded into the phloem?
- Which transporters are involved in feeding microbiota? How are their activities controlled? How can pathogen feeding be prevented?
- Do other efflux transporters serve as host susceptibility factors for pathogens?

## Funding

This research was supported by Deutsche Forschungsgemeinschaft (DFG, German Research Foundation) under Germany’s Excellence Strategy—EXC-2048/1—Project ID 390686111, Deutsche Forschungsgemeinschaft (DFG, German Research Foundation) SFB 1208—Project-ID 267205415, the Alexander von Humboldt Professorship, the National Science Foundation (SECRETome Project: Systematic Evaluation of CellulaR ExporT from plant cells, IOS-1546879) and the European Research Council (ERC) under the European Union’s Horizon 2020 research and innovation programme (grant agreement no. 951292, Sympore) to W.B.F.

*Conflict of interest statement*. None declared.

## Supplementary Material

kiab228_Supplementary_Data

## Notes

J.Y.K., W.B.F., and M.M.W. conceptualized the review. All authors contributed major sections of this manuscript and prepared specific figures and tables (J.Y.K.: scRNA-seq analysis, SWEETs, other efflux transporters, M.M.W.: UmamiTs, phylogenetic analysis, E.L.: other efflux transporters, symbiosis and microbiota sections, T.Y.P. and M.L.: metabolic gene expression analysis).

The author responsible for distribution of materials integral to the findings presented in this article in accordance with the policy described in the Instructions for Authors (https://academic.oup.com/plphys/pages/general-instructions) is: Michael M. Wudick (wudick@hhu.de).
