# Supplementary material for: Cellular export of sugars and amino acids: role in feeding other cells and organisms
Source: Plant Physiol. 2021 May 20;187(4):1893–914. doi: 10.1093/plphys/kiab228 (PMC8644676; doi:10.1093/plphys/kiab228)
Supplement: kiab228_Supplementary_Data [file kiab228_supplementary_data.pdf]

Supplemental Table S1. Gene IDs of SWEETs used for phylogenetic trees.

Gene annotation list for SWEETs from *Arabidopsis thaliana* (*At*), *Oryza sativa* (*Os*), *Volvox carteri f. nagariensis* (*Vc*), and *Zea mays* (*Zm*).

Gene name	ID
AtSWEET1	At1g21460
AtSWEET2	At3g14770
AtSWEET3	At5g53190
AtSWEET4	At3g28007
AtSWEET5	At5g62850
AtSWEET6	At1g66770
AtSWEET7	At4g10850
AtSWEET8	At5g40260
AtSWEET9	At2g39060
AtSWEET10	At5g50790
AtSWEET11	At3g48740
AtSWEET12	At5g23660
AtSWEET13	At5g50800
AtSWEET14	At4g25010
AtSWEET15	At5g13170
AtSWEET16	At3g16690
AtSWEET17	At4g15920

Gene name	ID
ZmSWEET1a	GRMZM2G039365
ZmSWEET1b	GRMZM2G153358
ZmSWEET2a	GRMZM2G324903
ZmSWEET2b	GRMZM6G056015
ZmSWEET3a	GRMZM2G179679
ZmSWEET3b	GRMZM2G060974
ZmSWEET4a	GRMZM2G000812
ZmSWEET4b	GRMZM2G144581
ZmSWEET4c	GRMZM2G137954
ZmSWEET6a	GRMZM2G157675
ZmSWEET6b	GRMZM2G416965
ZmSWEET11a	GRMZM2G368827
ZmSWEET11b	GRMZM5G872141
ZmSWEET12a	GRMZM2G133322
ZmSWEET12b	GRMZM2G099609
ZmSWEET13a	GRMZM2G173669
ZmSWEET13b	GRMZM2G021706
ZmSWEET13c	GRMZM2G179349
ZmSWEET14a	GRMZM2G094955
ZmSWEET14b	GRMZM2G015976
ZmSWEET15a	GRMZM2G168365
ZmSWEET15b	GRMZM5G872392
ZmSWEET16	GRMZM2G107597
ZmSWEET17a	GRMZM2G106462
ZmSWEET17b	GRMZM2G111926

Gene name	ID (MSU / RAP)
OsSWEET1a	LOC_Os01g65880/Os01g0881300
OsSWEET1b	LOC_Os05g35140/Os05g0426000
OsSWEET2a	LOC_Os01g36070/Os01g0541800
OsSWEET2b	LOC_Os01g50460/Os01g0700100
OsSWEET3a	LOC_Os05g12320/Os05g0214300
OsSWEET3b	LOC_Os01g12130/Os01g0220700
OsSWEET4	LOC_Os02g19820/Os02g0301100
OsSWEET5	LOC_Os05g51090/Os05g0588500
OsSWEET6a	LOC_Os01g42110/Os01g0606000
OsSWEET6b	LOC_Os01g42090/Os01g0605700
OsSWEET7a	LOC_Os09g08030/Os09g0254600
OsSWEET7b	LOC_Os09g08440/Os09g0258700
OsSWEET7c	LOC_Os12g07860/Os12g0178500
OsSWEET7d	LOC_Os09g08490/Os09g0259200
OsSWEET7e	LOC_Os09g08270/Os09g0256650
OsSWEET11a	LOC_Os08g42350/Os08g0535200
OsSWEET11b	LOC_Os05g35140/Os05g0426000
OsSWEET12	LOC_Os03g22590/Os03g0347500
OsSWEET13	LOC_Os12g29220/Os12g0476200
OsSWEET14	LOC_Os11g31190/Os11g0508600
OsSWEET15	LOC_Os02g30910/Os02g0513100
OsSWEET16	LOC_Os03g22200/Os03g0341300
VcSWEET	VOLCADRAFT_115772

Supplemental Table S2. Gene IDs of UmamiTs used for phylogenetic trees.
Gene annotation list for UmamiTs from *Arabidopsis thaliana* (*At*) and *Neisseria meningitidis* (*Nm*).

Gene name	ID	Gene name	ID
AtUmamiT1	At5g45370	***AtUmamiT25***	At1g09380
AtUmamiT2	At4g19185	***AtUmamiT26***	At1g11460
AtUmamiT3	At3g45870	***AtUmamiT27***	At1g11450
AtUmamiT4	At3g18200	***AtUmamiT28***	At1g01070
AtUmamiT5	At1g75500	***AtUmamiT29***	At4g01430
AtUmamiT6	At3g53210	***AtUmamiT30***	At4g01450
AtUmamiT7	At5g47470	***AtUmamiT31***	At4g01440
AtUmamiT8	At4g16620	***AtUmamiT32***	At3g30340
AtUmamiT9	At5g07050	***AtUmamiT33***	At4g28040
AtUmamiT10	At3g56620	***AtUmamiT34***	At4g30420
AtUmamiT11	At2g40900	***AtUmamiT35***	At1g60050
AtUmamiT12	At2g37460	***AtUmamiT36***	At1g70260
AtUmamiT13	At2g37450	***AtUmamiT37***	At5g40230
AtUmamiT14	At2g39510	***AtUmamiT38***	At4g15540
AtUmamiT15	At5g13670	***AtUmamiT40***	At5g40240
AtUmamiT16	At4g24980	***AtUmamiT41***	At3g28050
AtUmamiT17	At4g08300	***AtUmamiT42***	At5g40210
AtUmamiT18	At1g44800	***AtUmamiT43***	At3g28060
AtUmamiT19	At1g21890	***AtUmamiT44***	At3g28130
AtUmamiT20	At4g08290	***AtUmamiT45***	At3g28100
AtUmamiT21	At5g64700	***AtUmamiT46***	At3g28070
AtUmamiT22	At1g43650	***AtUmamiT47***	At3g28080
AtUmamiT23	At1g68170		
AtUmamiT24	At1g25270	***NmBAT***	WP_002217944.1

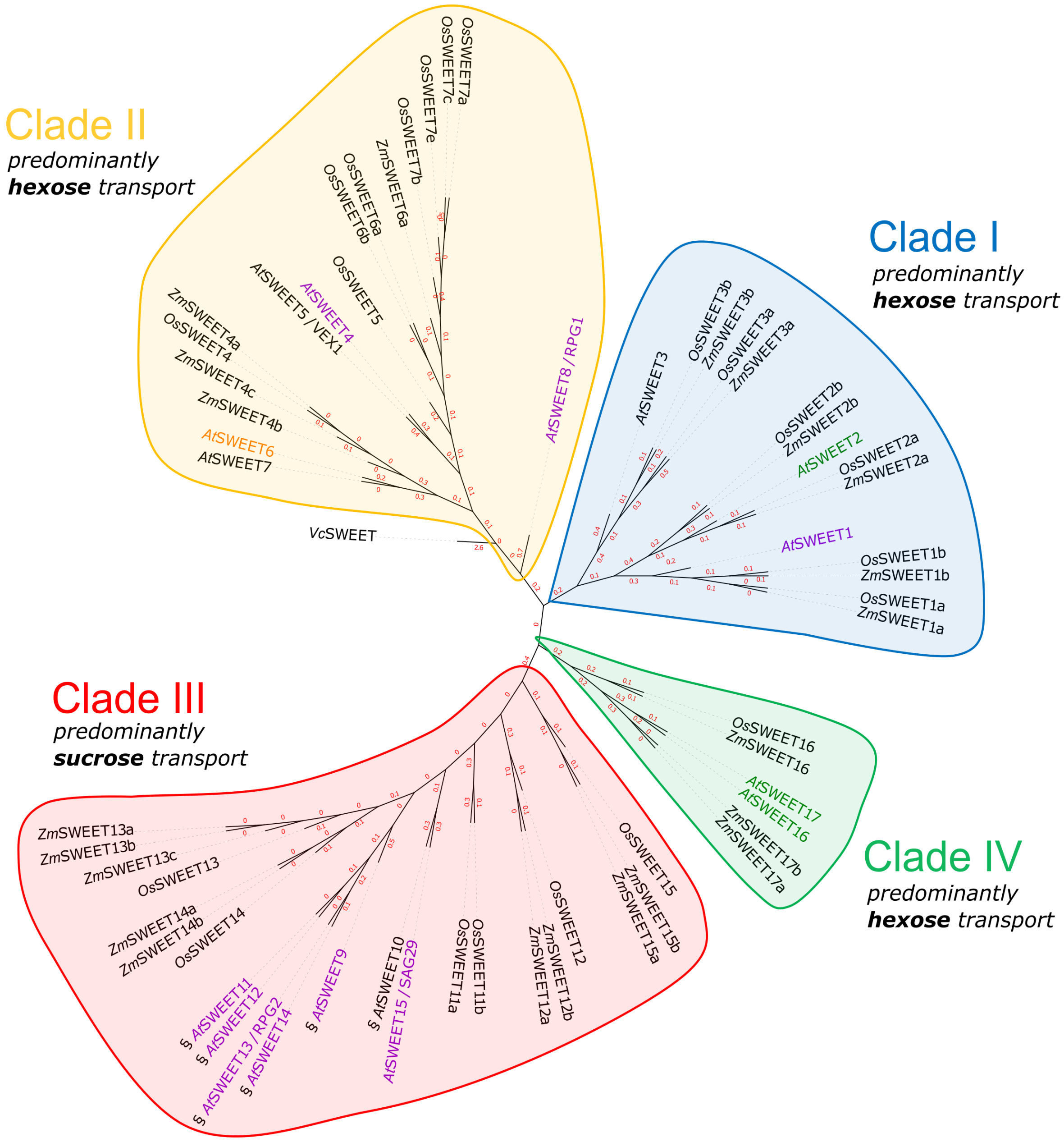

Supplemental Figure S1. Phylogenetic analysis of SWEET family proteins of *Arabidopsis* (*At*), *Oryza sativa* (*Os*, rice), and *Zea mays* (*Zm*, maize). A SWEET paralog from *Volvox carteri* (*Vc*) served as an outgroup. Protein sequences were obtained from Aramemnon (http://aramemnon.uni-koeln.de), UniProt (https://www.uniprot.org), and MaizeGDB (https://www.maizegdb.org). Gene IDs are provided in Supplementary Table S1. The unrooted tree was generated using the NGPhylogeny tool (https://ngphylogeny.fr) (Lemoine et al., 2019) and visualized with the help of iTOL (https://itol.embl.de) (Letunic and Bork, 2019). Only the conserved regions of the protein were considered for this analysis (the number of *Mt*N3/Saliva domains was predicted using AramTmCon (http://aramemnon.uni-koeln.de) and TMHMM (http://www.cbs.dtu.dk/services/TMHMM). Protein sequences were aligned using the MAFFT alignment program (Katoh et al., 2019) with a gap-opening penalty of 1.53 and a gap-extension penalty of 0.123. The phylogenetic tree was generated using the neighbor-joining method with the LG amino acid replacement matrix. Clade support scores were calculated by bootstrapping (*n* = 1,000 replicates); branch length values are displayed in red. Color codes reflect a reported localization: plasma membrane (purple), tonoplast (green), and ER (orange). Substrates are indicated, including gibberellic acid (§). Note that clade I, II and IV SWEETs mainly transport hexoses, and clade III SWEETs transport sucrose, but exceptions have been reported (see Table 2).

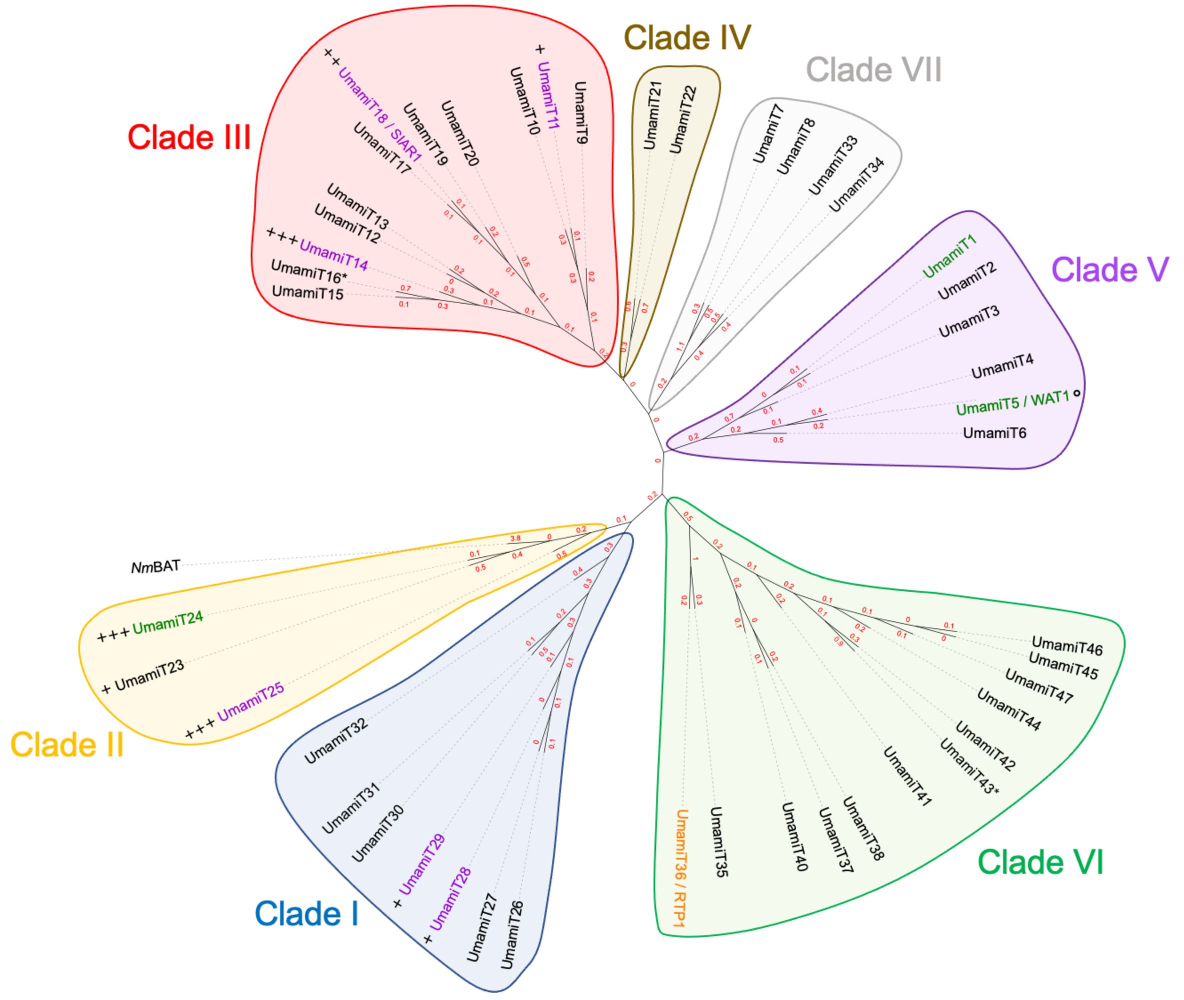

Supplemental Figure S2. The phylogeny of 46 members of the *A. thaliana* UmamiT family. The EamA family transporter from *N. meningitidis* (*Nm*BAT) was used as an outgroup (for gene IDs consult Supplementary Table S2). The tree is presented as an unrooted tree and was generated using the NGPhylogeny tool (https://ngphylogeny.fr) (Lemoine et al., 2019) and visualized with the help of iTOL (https://itol.embl.de) (Letunic and Bork, 2019). The highly variable N- and C-terminal protein sequences were truncated, and only the conserved regions of the protein were considered for this analysis. Protein sequences were aligned using the MAFFT alignment program (Katoh et al., 2019) with a gap-opening penalty of 1.53 and a gap-extension penalty of 0.123. The phylogenetic tree was generated using the neighbor-joining method with the LG amino acid replacement matrix. Clade support scores were calculated by bootstrapping (n = 1,000 replicates) and branch length values are displayed in red. Color codes reflect a reported localization in the plasma membrane (purple), the tonoplast (green), and ER (orange). Asterisks indicate putative semiUmamiTs. The number of transported substrates is indicated as follows: 1-5 amino acids (+), 6-10 amino acids (++), >10 amino acids (+++), transport of auxin (◯), see Table 3. Clade annotation is based on (Ladwig et al., 2012) with the addition of a previously non-annotated new clade (Clade VII).
